# Supplementary material for: Identification of photoperiod-induced specific miRNAs in the adrenal glands of Sunite sheep (Ovis aries)
Source: Front Vet Sci. 2022 Jul 22;9:888207. doi: 10.3389/fvets.2022.888207 (PMC9354845; doi:10.3389/fvets.2022.888207)
Supplement: Supplementary file 1 [file Data_Sheet_1.ZIP › Supplementary file/miRDeep2 file.zip!/miRDeep2 file/Novel_Structure/LP42A1/Novel_11.pdf]

Provisional ID	:	NC_019461.2_10415
Score total	:	2
Score for star read(s)	:	3.9
Score for read counts	:	-3.9
Score for mfe	:	0.4
Score for randfold	:	1.6
Score for cons. seed	:	
Total read count	:	4
Mature read count	:	3
Loop read count	:	0
Star read count	:	1

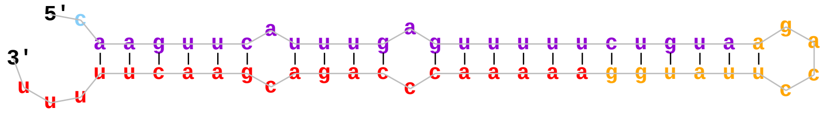

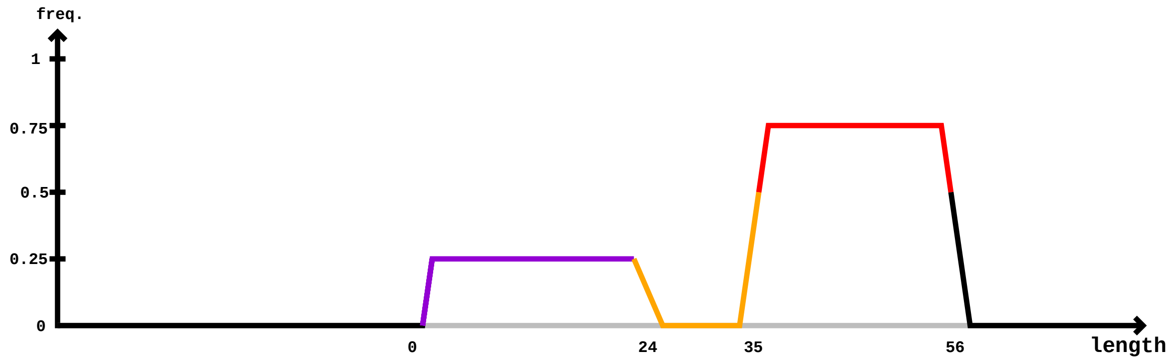

Star Mature

```
5'- uacauagaaaauggaauauauuguuccauuauuguucaaguucauuugaguuuuucuguaagaccuuauggaaaaacccagacgaacuuuuuggcacaaauguaaacguua   -3'    obs
    uacauagaaaauggaauauauuguuccauuauuguucaaguucauuugaguuuuucuguaagaccuuauggaaaaacccagacgaacuuuuuggcacaaauguaaacguua          exp
    (((((....(((((((((...)))))))))..((((.((((((.((((.((((((((((((....)))))))))))))).)))).))))))...))))...))))).......    reads      mm        sample
    .....................................aaguucauuugagCuuuucugua...................................................    1          1         seq
    ......................................................................gaaaaacccagacgaacuuuu....................    1          0         seq
    .......................................................................aaaaacccagacgaacuuuuu...................    1          0         seq
    ........................................................................aaaacccaAacgaacuuuuug..................    1          1         seq
```
